# New York Letter

**Published:** 1902-06

**Authors:** 


### Domestic Correspondence.

### NEW YORK LETTER.

To the Editor:

Sir,—The regular monthly meeting of The New York Institute of Stomatology was held at the office of Dr. S. E. Davenport, on Tuesday, April 1. That all-important topic, "Orthodontia," was the paper for the evening.

The paper, "Stray Thoughts about Regulating," was presented by S. E. Davenport, M.D., D.D.S., of New York City. He presented in a clear and concise manner his method and the advantages which he claimed for it, also other important points bearing upon the subject.

Primarily his appliances are made removable. As to the arguments advanced for and against removable appliances in regulating, the doctor dwelt at some length on his method, consisting mostly of rubber plates with springy German silver wire attachments, and in some indicated instances he uses screws or other methods. He touched upon the importance of properly diagnosing each case, of taking impressions, of studying the casts and comparing them with the patient's face before fully deciding upon the course to pursue. The principle of separating teeth by the fish-line method he discussed, the manner of adjusting it, and its relative value in regulating. He considered the desirability of avoiding extraction in nearly every instance; clearly expressing reasons for the position taken by him. For, as he said, without extraction we have, in the crowded arch, only to expand the arch and rearrange the teeth, while extraction makes the condition at once abnormal.

He discussed his methods of expanding the upper and lower arches, and many other points of such great practical value that the careful study of this important paper is earnestly recommended. He gave credit to Dr. V. H. Jackson for systematizing the use of wire cribs, and also for his many valuable contributions on orthodontia.

Dr. J. N. Farrar, of New York City, discussed the paper. Among other things stated was that of advocating extraction in indicated cases. Drs. Littig, Kimball, Gillett, and Bogue, of New York, and Ames, of Boston, were among those who joined in the interesting discussion that followed. The many present listened to it with great interest, and felt more than repaid for having been so fortunate as to be present at the reading and discussion of so valuable a contribution to dental literature.

Under incidents of office practice, which preceded the paper of the evening, Dr. Wilson presented a very interesting case of the retarding of the upper central and lateral incisors by a deciduous tooth, which he discovered, by means of the radiograph, embedded in the jaw-bone, and, the obstruction being removed, the doctor hopes to eventually bring the teeth into their relative proper positions.

On Tuesday, April 8, the regular monthly meeting of the First

District Dental Society was held, the evening being devoted to the election of officers for the ensuing year, and also the reading of the annual reports, which were very favorable indeed.

Those who heard Dr. Charles McManus, of Hartford, read a paper on "The Makers of Dentistry," before the New York Odontological Society, on April 15, will ever remember it as intensely interesting and instructive, and the great value of the paper is at once apparent from the fact that after its close Dr. S. G. Perry moved that copies of it be printed and distributed.

With the stereopticon slides Dr. McManus threw upon the screen the portraits of the makers of dentistry, commencing with Ambroise Paré, "the barber surgeon," who died about 1790, and who really was one of the founders of dentistry. He displayed portraits of a long line of illustrious men who were instrumental in developing and promoting the very best and highest interests of our profession. It is a work that should be duly printed and adopted as a text-book by all the dental colleges for students, and even older practitioners, who after appreciating the many trials which the founders endured are bound to receive some inspiration from the work.

Among other points brought out was that of an expensive edition that was published by Fouchard in 1728; of how John Hunter, an Englishman, practised about a century ago; of the first dental company formed in this country for the practice of dentistry, started in Boston in 1636, and known as "The Plymouth Company;" of how John Mills started practising in New York City in 1735; of how Joseph Lemaire, a young French army officer, practised in and about the army during the Revolutionary War; of James Gardette, who was the first to use gold-foil; of John Greenwood, Washington's dentist; of J. R. Spooner, the first to use arsenic in 1836; of Dr. H. H. Hayden, one of "the noblest of them all," who did so much to further the interests of dentistry; of Drs. C. A. Harris, Horace Wells, Dr. Riggs; of Dr. Arthur, one of the first to advocate the annealing of gold-foil; of Dr. J. B. Morrison, of dental engine fame, and many others. In each instance Dr. McManus gave a brief biography, and brought out most admirably the respective dominant characteristics of each dentist.

The afternoon clinic was also well attended, and many valuable practical illustrations were given. Among those that might be mentioned were Dr. W. T. Shields's method of utilizing saddle-back teeth in the construction of æsthetic bridge-work and Dr. R. L. Watkins describing the method of examination of fresh specimens of the human blood with the microscope, for the purpose of diagnosing certain abnormal conditions of the system, some of which are existent in cases of pyorrhœa-alveolaris.

"LOCHINVAR."

